# Topology of the Electron Density and of Its Laplacian from Periodic LCAO Calculations on *f*-Electron Materials: The Case of Cesium Uranyl Chloride

**DOI:** 10.3390/molecules26144227

**Published:** 2021-07-12

**Authors:** Alessandro Cossard, Silvia Casassa, Carlo Gatti, Jacques K. Desmarais, Alessandro Erba

**Affiliations:** 1 Dipartimento di Chimica, Università di Torino, Via Giuria 5, 10125 Torino, Italy; alessandro.cossard@unito.it (A.C.); silvia.casassa@unito.it (S.C.); 2 CNR-SCITEC, Istituto di Scienze e Tecnologie Chimiche “Giulio Natta”, Via C. Golgi 19, 20133 Milano, Italy; Carlo.Gatti@scitec.cnr.it

**Keywords:** chemical bonding, actinides, Topond program

## Abstract

The chemistry of *f*-electrons in lanthanide and actinide materials is yet to be fully rationalized. Quantum-mechanical simulations can provide useful complementary insight to that obtained from experiments. The quantum theory of atoms in molecules and crystals (QTAIMAC), through thorough topological analysis of the electron density (often complemented by that of its Laplacian) constitutes a general and robust theoretical framework to analyze chemical bonding features from a computed wave function. Here, we present the extension of the Topond module (previously limited to work in terms of *s*-, *p*- and *d*-type basis functions only) of the Crystal program to *f*- and *g*-type basis functions within the linear combination of atomic orbitals (LCAO) approach. This allows for an effective QTAIMAC analysis of chemical bonding of lanthanide and actinide materials. The new implemented algorithms are applied to the analysis of the spatial distribution of the electron density and its Laplacian of the cesium uranyl chloride, Cs${}_{2}$UO${}_{2}$Cl${}_{4}$, crystal. Discrepancies between the present theoretical description of chemical bonding and that obtained from a previously reconstructed electron density by experimental X-ray diffraction are illustrated and discussed.

## 1. Introduction

The degree of participation and covalency of 4*f* and 5*f* electrons in the chemical bonding of lanthanide and actinide complexes and materials (including oxides) is an intriguing topic in chemistry [1,2,3,4,5,6,7], with both fundamental and technological implications. Indeed, the renewed interest in the nuclear power industry and nuclear waste management has put the separation process of uranium from lanthanides and other minor actinides under the spotlight. The efficiency and selectivity of the process depends on the respective bond strength [8,9]. In particular, several factors (including strong relativistic effects, weak crystal fields and strong electron correlation) contribute to the occurrence of a composite valence manifold (comprising of 5*f*, 6*p*, 6*d* and 7*s* orbital shells) in actinide–ligand bonds [5,10,11,12].

The quantum theory of atoms in molecules and crystals (QTAIMAC) provides a rigorous formal framework within which multiple aspects of chemical bonding can be analyzed [13,14]. The theory builds on a topological analysis of the electron density ρ(r), which can be either determined by theoretical calculations or measured by experimental X-ray diffraction. In both cases, several technical challenges need to be overcome for effective treatment of *f*-electrons, to be briefly addressed below. Such an approach is often complemented by an analysis of the topology of the Laplacian of the density $\nabla^{2}\rho \left(r\right)$, which provides additional information on the spatial distribution of the electrons and, in particular, on the asphericity of (bonded) atoms [15].

So far, the electron density of very few actinide materials could be successfully reconstructed from X-ray diffraction measurements: Th(S${}_{2}$PMe${}_{2}$)${}_{4}$, cesium uranyl chloride Cs${}_{2}$UO${}_{2}$Cl${}_{4}$ and tetraphenyl phosphate uranium hexafluoride [PPh${}_{4}$][UF${}_{6}$] [16,17,18,19,20]. In particular, significant advances have been recently reported by Pinkerton and co-workers on data collection and analysis, which finally allow for the accurate experimental determination of the electron density of actinide materials [21]. Similarly, from a theoretical perspective, the many challenges related to a proper and simultaneous description of relativistic effects of core electrons and electron correlation of valence electrons (including *f*-type ones) made it possible to perform a QTAIM analysis on top of computed electron densities of actinide molecular complexes only recently [22,23,24,25,26,27,28,29].

In this paper, we present recent formal and software advances in the quantum-mechanical calculation of the electron density and its Laplacian as well as in their topological analyses, for *f*-electron materials, within periodic boundary conditions for an effective study of chemical bonding in extended systems. In our methodology, crystalline orbitals are expressed as linear combinations of atomic orbitals (LCAO). Quantum-mechanical calculations are performed with a developmental version of the Crystal program [30,31], where the LCAO approach has recently been extended to *g*-type basis functions [32,33]. The topological analysis of the electron density ρ(r) and its Laplacian $\nabla^{2}\rho \left(r\right)$ is performed with a developmental version of the Topond module [14,34,35], which can be run in parallel with a high efficiency [36]. Here, we discuss the strategy that we have followed to extend this module (formerly limited to work in terms of *s*-, *p*- and *d*-type basis functions only) to *f*- and *g*-type basis functions as well. The new implementation has already been successfully applied to the study of chemical bonding in the [PPh${}_{4}$][UF${}_{6}$] crystal, where the topology of the computed density and its Laplacian was found to match that from the experiment very satisfactorily [37]. In this paper, we study the topology of ρ(r) and $\nabla^{2}\rho \left(r\right)$ of the cesium uranyl chloride Cs${}_{2}$UO${}_{2}$Cl${}_{4}$ crystal. The electron density of this system has been reconstructed experimentally and its topology analyzed with the QTAIMAC [18,19]. A detailed theoretical investigation of the electron distribution and chemical bonding in the [UO${}_{2}$Cl${}_{4}$]${}^{2-}$ molecular fragment has been performed, which described the weakening of the U–O uranyl bond due to the equatorial ligands [38]. The computed values of ρ(r) and $\nabla^{2}\rho \left(r\right)$ at the U–Cl bond critical points were in excellent agreement with those from the experiments while a somewhat looser agreement was reported for the U–O bond. A subsequent study investigated whether the observed discrepancy could be due to missing environmental effects in the simulations and concluded that these are very small and therefore cannot explain the observed differences [39].

## 2. Computational Details

Calculations on the molecular fragment [UO${}_{2}$Cl${}_{4}$]${}^{2-}$ and extended periodic crystal Cs${}_{2}$UO${}_{2}$Cl${}_{4}$ are performed with the Crystal program by use of the global hybrid B3LYP exchange-correlation functional [40] of the density functional theory (DFT). In the periodic calculations, reciprocal space is sampled on a regular 6×6×6 Monkhorst-Pack grid, corresponding to 68 **k**-points in the symmetry-irreducible Brillouin zone. Scalar relativistic effects of U must be accounted for [27,28,29,41] and, here, are described by use of small-core effective pseudopotentials, ECP60MDF (with 60 electrons in the core for U) [42,43]. The valence of U is described by a (10*s*9*p*7*d*5*f*1*g*)/[10*s*9*p*7*d*5*f*1*g*] basis set: we indicate within round brackets the number of Gaussian primitive functions used for the various angular quantum numbers and within square brackets the number of shells in which they are contracted. In this case, we use a fully uncontracted basis. With respect to the original basis set optimized for molecular calculations, some very diffuse exponents have been removed (crucially the most diffuse *p*-type exponent) that were causing linear dependencies in the periodic calculations. While the program has recently been extended to the treatment of spin-orbit coupling [44,45,46,47], this relativistic effect is disregarded here. This is because, while making the calculations significantly more demanding, it has been previously shown to induce minor changes to chemical bonding for such systems [48]. Oxygen and chlorine are described by molecular def2-TZVP basis sets of (11*s*6*p*2*d*1*f*)/[5*s*3*p*2*d*1*f*] and (14*s*9*p*3*d*1*f*)/[5*s*5*p*2*d*1*f*] type, respectively [49]. For Cs, Hay–Wadt small-core pseudopotentials are used [50] in combination with a (4*s*4*p*1*d*)/[2sp1*d*] valence.

## 3. The Implementation

In this section, we present some formal aspects related to the implemented algorithms for the extension to *f*- and *g*-type basis functions of the topological analysis of the electron density and its Laplacian in the Topond program module.

### 3.1. Basis Functions

In the periodic LCAO framework within the Crystal and Topond programs, the one-electron crystalline orbitals (COs) are expressed as a linear combination of Bloch functions $\phi_{\mu }\left(r,k\right)$, depending on the spatial coordinates r of an electron and its wave vector k. A Bloch function is written, in turn, as a Fourier transform of the associated atomic orbital (AO) $\chi_{\mu }\left(r\right)$:

(1) $$\phi_{\mu }\left(r,k\right)=\sum_{g}\chi_{\mu }\left(r-A_{\mu }-g\right)e^{ik\cdot g}\;,$$

where the sum extends to the infinite set of lattice vectors g and $A_{\mu }$ is the position of the atom on which $\chi_{\mu }$ is centered in the reference cell. In Crystal, the AOs are expressed as a linear combination of normalized real-solid spherical harmonic Gaussian-type functions (RSSH-GTF) $R_{n,l,m}\left(r;\alpha_{j}^{\lambda }\right)$:

(2) $$\chi_{\mu }\left(r\right)=\sum_{j\in \lambda }d_{j}^{\lambda }R_{n,l,m}\left(r;\alpha_{j}^{\lambda }\right)\;,$$

where:

(3) $$R_{n,l,m}\left(r;\alpha_{j}^{\lambda }\right)=N_{\lambda }N_{l,m}N_{l}\left(\alpha_{j}^{\lambda }\right)X_{n,l,m}\left(r\right)e^{-\alpha_{j}^{\lambda }{∥r∥}^{2}}\;,$$

and $∥\cdots ∥$ is the Euclidean norm. In Equation (2), $d_{j}^{\lambda }$ and $\alpha_{j}^{\lambda }$ are fixed linear coefficients and exponents associated to a shell λ. In the context of the shell strategy, AOs with similar quantum numbers *n* and *l* but different *m* (e.g., the $2p_{x}$, $2p_{y}$ and $2p_{z}$ AOs) share a common set of $d_{j}^{\lambda }$ and $\alpha_{j}^{\lambda }$. Thus, formally, we may write $\lambda =\lambda \left(n,l\right)$, although the dependence of λ on the set of quantum numbers has been suppressed in Equations (2) and (3) to simplify the notation. $N_{\lambda }$, $N_{l}\left(\alpha_{j}^{\lambda }\right)$ and $N_{l,m}$ are normalization constants. The shell normalization constant $N_{\lambda }$ reads:

(4) $$N_{\lambda }={\left[\sum_{i,j\in \lambda }d_{i}^{\lambda }d_{j}^{\lambda }{\left(\frac{\sqrt{\alpha_{i}^{\lambda }\alpha_{j}^{\lambda }}}{\alpha_{i}^{\lambda }+\alpha_{j}^{\lambda }}\right)}^{l+3/2}\right]}^{-1/2}\;.$$

The *m*-dependent normalization constant $N_{l,m}$ reads:

(5) $$N_{l,m}={\left[\frac{\left(2l-1\right)!!\left(l+|m|\right)!}{2^{l}\left(2-\delta_{m,0}\right)\left(l-|m|\right)!}\right]}^{-1/2}\;,$$

where $\left(\cdots \right)!$ and $\left(\cdots \right)!!$ are the factorial and double factorial symbols and $\delta_{m,0}$ is the Kronecker delta returning 1 when m=0 and 0 when m≠0. For $N_{l}\left(\alpha_{j}^{\lambda }\right)$ we have:

(6) $$N_{l}\left(\alpha_{j}^{\lambda }\right)={\left[\frac{\pi^{3/2}}{{\left(2\alpha_{j}^{\lambda }\right)}^{l+3/2}}\right]}^{-1/2}\;.$$

Finally, in Equation (3) $X_{n,l,m}\left(r\right)$ is a RSSH, which may be expressed as a homogeneous polynomial of the Cartesian components $r_{x}$, $r_{y}$ and $r_{z}$ of the electron coordinates:

(7) $$X_{n,l,m}\left(r\right)={\left.\sum_{t,u,v}\right.}^{\prime }D_{l,m}\left(t,u,v\right)r_{x}^{t}r_{y}^{u}r_{z}^{v}\;,$$

in which $D_{l,m}\left(t,u,v\right)$ are linear coefficients and the sum in Equation (7) extends to all integer triplets t,u,v whose values are constrained by the equality t+u+v=2n+l. In the Crystal and Topond programs, the AOs in the basis set are defined using only RSSH-GTFs with null principal quantum number, n=0. However, RSSH-GTFs with n≠0 are still required in the Crystal program because they are used as auxiliary functions for the evaluation of electronic kinetic energy integrals.

### 3.2. The Electron Density and Its Derivatives

The QTAIMAC is based on a topological analysis of the electron density $\rho \left(r\right)$ (a scalar field), which requires the evaluation of its first- and second-order derivatives w.r.t. spatial coordinates in the definition of the gradient vector and Hessian matrix:

(8) $$\nabla \rho \left(r\right)=\left(\begin{array}{c} \frac{\partial \rho }{\partial r_{x}}\\ \frac{\partial \rho }{\partial r_{y}}\\ \frac{\partial \rho }{\partial r_{z}} \end{array}\right)\;H\left(r\right)=\left(\begin{array}{ccc} \frac{\partial^{2}\rho }{\partial r_{x}^{2}} & \frac{\partial^{2}\rho }{\partial r_{x}\partial r_{y}} & \frac{\partial^{2}\rho }{\partial r_{x}\partial r_{z}}\\ \frac{\partial^{2}\rho }{\partial r_{y}\partial r_{x}} & \frac{\partial^{2}\rho }{\partial r_{y}^{2}} & \frac{\partial^{2}\rho }{\partial r_{y}\partial r_{z}}\\ \frac{\partial^{2}\rho }{\partial r_{z}\partial r_{x}} & \frac{\partial^{2}\rho }{\partial r_{z}\partial r_{y}} & \frac{\partial^{2}\rho }{\partial r_{z}^{2}} \end{array}\right)$$

The trace of the Hessian matrix defines the Laplacian of the density (another scalar field):

(9) $$\nabla^{2}\rho \left(r\right)=\frac{\partial^{2}\rho }{\partial r_{x}^{2}}+\frac{\partial^{2}\rho }{\partial r_{y}^{2}}+\frac{\partial^{2}\rho }{\partial r_{z}^{2}}$$

The description of chemical bonding features passes through the identification of critical points (CPs) of the density, defined as those points in space $r_{\mathrm{CP}}$ where the gradient vector of the density vanishes $\nabla \rho \left(r_{\mathrm{CP}}\right)=0$. CPs can be classified into different types according to the signs of the corresponding eigenvalues of the Hessian matrix. Further information on the spatial distribution of the electrons can be obtained from a topological analysis of the Laplacian, whose positive and negative values correspond to regions of (relative) charge concentration and depletion, respectively. Valence shell charge concentrations (VSCCs) are particularly relevant to the rationalization of chemical bonding, and can be analyzed in terms of CPs of the Laplacian of type (3,+3), i.e., minima. It is often preferable to flip the sign of the Laplacian and work in terms of the following function:

(10) $$L\left(r\right)=-\nabla^{2}\rho \left(r\right)\;,$$

so that positive values and maxima of this function correspond to regions of charge concentration. Critical points of L(r) can be found and characterized by analyzing its first- and second-order derivatives (involving third- and fourth-order derivatives of the electron density, respectively).

Thus, at the heart of the Topond program lies the evaluation of the electron density $\rho \left(r\right)$ and of its derivatives w.r.t. Cartesian components of the spatial coordinate. In the following, $i,j,k,l\in \left[x,y,z\right]$ indices will be used to refer to Cartesian components. The density is defined as follows in the basis of AOs, using the shorthand notation $r_{\mu }=r-A_{\mu }$ and $r_{\nu }^{g}=r-A_{\nu }-g$:

(11) $$\rho \left(r\right)=\sum_{\mu \nu }\sum_{g}P_{\mu \nu }^{g}\chi_{\mu }\left(r_{\mu }\right)\chi_{\nu }\left(r_{\nu }^{g}\right)\;,$$

where $P_{\mu \nu }^{g}$ is the direct-space one-electron density matrix. For the first derivatives:

(12) $$\begin{array}{ccc} \frac{\partial \rho \left(r\right)}{\partial r_{i}} & = & \sum_{\mu \nu }\sum_{g}P_{\mu \nu }^{g}\left\{\left[\frac{\partial }{\partial r_{i}}\chi_{\mu }\left(r_{\mu }\right)\right]\chi_{\nu }\left(r_{\nu }^{g}\right)+\chi_{\mu }\left(r_{\mu }\right)\left[\frac{\partial }{\partial r_{i}}\chi_{\nu }\left(r_{\nu }^{g}\right)\right]\right\}\;. \end{array}$$

For the second derivatives:

(13) $$\begin{array}{ccc} \frac{\partial^{2}\rho \left(r\right)}{\partial r_{i}\partial r_{j}} & = & \sum_{\mu \nu }\sum_{g}P_{\mu \nu }^{g}\{\left[\frac{\partial^{2}}{\partial r_{i}\partial r_{j}}\chi_{\mu }\left(r_{\mu }\right)\right]\chi_{\nu }\left(r_{\nu }^{g}\right)+\chi_{\mu }\left(r_{\mu }\right)\left[\frac{\partial^{2}}{\partial r_{i}\partial r_{j}}\chi_{\nu }\left(r_{\nu }^{g}\right)\right]\\ & + & \left[\frac{\partial }{\partial r_{i}}\chi_{\mu }\left(r_{\mu }\right)\right]\left[\frac{\partial }{\partial r_{j}}\chi_{\nu }\left(r_{\nu }^{g}\right)\right]+\left[\frac{\partial }{\partial r_{j}}\chi_{\mu }\left(r_{\mu }\right)\right]\left[\frac{\partial }{\partial r_{i}}\chi_{\nu }\left(r_{\nu }^{g}\right)\right]\}\;. \end{array}$$

For the third derivatives:

(14) $$\begin{array}{ccc} \frac{\partial^{3}\rho \left(r\right)}{\partial r_{i}\partial r_{j}\partial r_{k}} & = & \sum_{\mu \nu }\sum_{g}P_{\mu \nu }^{g}\{\left[\frac{\partial^{3}}{\partial r_{i}\partial r_{j}\partial r_{k}}\chi_{\mu }\left(r_{\mu }\right)\right]\chi_{\nu }\left(r_{\nu }^{g}\right)+\chi_{\mu }\left(r_{\mu }\right)\left[\frac{\partial^{3}}{\partial r_{i}\partial r_{j}\partial r_{k}}\chi_{\nu }\left(r_{\nu }^{g}\right)\right]\\ \; & + & \left[\frac{\partial^{2}}{\partial r_{i}\partial r_{j}}\chi_{\mu }\left(r_{\mu }\right)\right]\left[\frac{\partial }{\partial r_{k}}\chi_{\nu }\left(r_{\nu }^{g}\right)\right]+\left[\frac{\partial^{2}}{\partial r_{i}\partial r_{k}}\chi_{\mu }\left(r_{\mu }\right)\right]\left[\frac{\partial }{\partial r_{j}}\chi_{\nu }\left(r_{\nu }^{g}\right)\right]\\ \; & + & \left[\frac{\partial^{2}}{\partial r_{j}\partial r_{k}}\chi_{\mu }\left(r_{\mu }\right)\right]\left[\frac{\partial }{\partial r_{i}}\chi_{\nu }\left(r_{\nu }^{g}\right)\right]+\left[\frac{\partial }{\partial r_{i}}\chi_{\mu }\left(r_{\mu }\right)\right]\left[\frac{\partial^{2}}{\partial r_{j}\partial r_{k}}\chi_{\nu }\left(r_{\nu }^{g}\right)\right]\\ \; & + & \left[\frac{\partial }{\partial r_{j}}\chi_{\mu }\left(r_{\mu }\right)\right]\left[\frac{\partial^{2}}{\partial r_{i}\partial r_{k}}\chi_{\nu }\left(r_{\nu }^{g}\right)\right]+\left[\frac{\partial }{\partial r_{k}}\chi_{\mu }\left(r_{\mu }\right)\right]\left[\frac{\partial^{2}}{\partial r_{i}\partial r_{j}}\chi_{\nu }\left(r_{\nu }^{g}\right)\right]\}\;, \end{array}$$

and for the fourth derivatives:

(15) $$\begin{array}{ccc} \frac{\partial^{4}\rho \left(r\right)}{\partial r_{i}\partial r_{j}\partial r_{k}\partial r_{l}} & = & \sum_{\mu \nu }\sum_{g}P_{\mu \nu }^{g}\{\left[\frac{\partial^{4}}{\partial r_{i}\partial r_{j}\partial r_{k}\partial r_{l}}\chi_{\mu }\left(r_{\mu }\right)\right]\chi_{\nu }\left(r_{\nu }^{g}\right)+\chi_{\mu }\left(r_{\mu }\right)\left[\frac{\partial^{4}}{\partial r_{i}\partial r_{j}\partial r_{k}\partial r_{l}}\chi_{\nu }\left(r_{\nu }^{g}\right)\right]\\ \; & + & \left[\frac{\partial^{3}}{\partial r_{i}\partial r_{j}\partial r_{k}}\chi_{\mu }\left(r_{\mu }\right)\right]\left[\frac{\partial }{\partial r_{l}}\chi_{\nu }\left(r_{\nu }^{g}\right)\right]+\left[\frac{\partial^{3}}{\partial r_{i}\partial r_{j}\partial r_{l}}\chi_{\mu }\left(r_{\mu }\right)\right]\left[\frac{\partial }{\partial r_{k}}\chi_{\nu }\left(r_{\nu }^{g}\right)\right]\\ \; & + & \left[\frac{\partial^{3}}{\partial r_{i}\partial r_{k}\partial r_{l}}\chi_{\mu }\left(r_{\mu }\right)\right]\left[\frac{\partial }{\partial r_{j}}\chi_{\nu }\left(r_{\nu }^{g}\right)\right]+\left[\frac{\partial^{3}}{\partial r_{j}\partial r_{k}\partial r_{l}}\chi_{\mu }\left(r_{\mu }\right)\right]\left[\frac{\partial }{\partial r_{i}}\chi_{\nu }\left(r_{\nu }^{g}\right)\right]\\ \; & + & \left[\frac{\partial^{2}}{\partial r_{i}\partial r_{j}}\chi_{\mu }\left(r_{\mu }\right)\right]\left[\frac{\partial^{2}}{\partial r_{k}\partial r_{l}}\chi_{\nu }\left(r_{\nu }^{g}\right)\right]+\left[\frac{\partial^{2}}{\partial r_{i}\partial r_{k}}\chi_{\mu }\left(r_{\mu }\right)\right]\left[\frac{\partial^{2}}{\partial r_{j}\partial r_{l}}\chi_{\nu }\left(r_{\nu }^{g}\right)\right]\\ \; & + & \left[\frac{\partial^{2}}{\partial r_{i}\partial r_{l}}\chi_{\mu }\left(r_{\mu }\right)\right]\left[\frac{\partial^{2}}{\partial r_{j}\partial r_{k}}\chi_{\nu }\left(r_{\nu }^{g}\right)\right]+\left[\frac{\partial^{2}}{\partial r_{j}\partial r_{k}}\chi_{\mu }\left(r_{\mu }\right)\right]\left[\frac{\partial^{2}}{\partial r_{i}\partial r_{l}}\chi_{\nu }\left(r_{\nu }^{g}\right)\right]\\ \; & + & \left[\frac{\partial^{2}}{\partial r_{j}\partial r_{l}}\chi_{\mu }\left(r_{\mu }\right)\right]\left[\frac{\partial^{2}}{\partial r_{i}\partial r_{k}}\chi_{\nu }\left(r_{\nu }^{g}\right)\right]+\left[\frac{\partial^{2}}{\partial r_{l}\partial r_{k}}\chi_{\mu }\left(r_{\mu }\right)\right]\left[\frac{\partial^{2}}{\partial r_{i}\partial r_{j}}\chi_{\nu }\left(r_{\nu }^{g}\right)\right]\\ \; & + & \left[\frac{\partial }{\partial r_{i}}\chi_{\mu }\left(r_{\mu }\right)\right]\left[\frac{\partial^{3}}{\partial r_{j}\partial r_{k}\partial r_{l}}\chi_{\nu }\left(r_{\nu }^{g}\right)\right]+\left[\frac{\partial }{\partial r_{j}}\chi_{\mu }\left(r_{\mu }\right)\right]\left[\frac{\partial^{3}}{\partial r_{i}\partial r_{k}\partial r_{l}}\chi_{\nu }\left(r_{\nu }^{g}\right)\right]\\ \; & + & \left[\frac{\partial }{\partial r_{k}}\chi_{\mu }\left(r_{\mu }\right)\right]\left[\frac{\partial^{3}}{\partial r_{i}\partial r_{j}\partial r_{l}}\chi_{\nu }\left(r_{\nu }^{g}\right)\right]+\left[\frac{\partial }{\partial r_{l}}\chi_{\mu }\left(r_{\mu }\right)\right]\left[\frac{\partial^{3}}{\partial r_{i}\partial r_{j}\partial r_{k}}\chi_{\nu }\left(r_{\nu }^{g}\right)\right]\}\;, \end{array}$$

From inspection of the equations above, we see that the evaluation of the electron density and/or of its derivatives boils down to the efficient evaluation of pair products of two AOs and/or pair products of their derivatives. Bearing in mind the expansion in Equations (2) and (3), the algorithms implemented in the Topond module rely on the efficient evaluation of pair products of RSSH-GTFs and/or of their derivatives.

### 3.3. Old Strategy Based on the Expansion in Hermite Gaussian Type Functions

The previously existing strategy implemented in the Topond module by Victor R. Saunders (limited to *s*, *p* and *d* AOs) was based on the expansion of any RSSH-GTF pair product into an auxiliary basis [51]:

(16) $$\begin{array}{c} R_{n,l,m}\left(\alpha ,r-A\right)R_{\tilde{n},\tilde{l},\tilde{m}}\left(\beta ,r-B\right)={\left.\sum_{t,u,v}\right.}^{\prime \prime }E\left[n,l,m,\tilde{n},\tilde{l},\tilde{m},t,u,v\right]\Lambda \left(\gamma ,r-P,t,u,v\right)\;, \end{array}$$

where the $E\left[\cdots \right]$ are linear coefficients and the Λs are Hermite Gaussian-type functions (HGTF):

(17) $$\Lambda \left(\gamma ,r-P,t,u,v\right)={\left(\frac{\partial }{\partial P_{x}}\right)}^{t}{\left(\frac{\partial }{\partial P_{y}}\right)}^{u}{\left(\frac{\partial }{\partial P_{z}}\right)}^{v}e^{-\gamma {∥r-P∥}^{2}}\;.$$

In Equation (16), the values of γ and P are obtained from the Gaussian product theorem (i.e., γ=α+β and P=(αA+βB)/γ and the sum over the indices t,u,v is extended to all integer triplets that satisfy the inequality $t+u+v\leq 2n+2\tilde{n}+l+\tilde{l}$. The $E\left[\cdots \right]$ coefficients can be conveniently generated from recurrence relations [51]. Moreover, once these coefficients are generated for a product pair of RSSH-GTFs with a given set of quantum numbers, they can be re-used to generate the expressions for those product pairs of RSSH-GTFs with lower quantum numbers but involving derivatives, which results in a general and efficient algorithm. However, let us note that the recurrence relations were never coded as such and instead the explicit expressions for all the relevant $E\left[\cdots \right]$ coefficients were pre-determined and hard-coded. While making the implementation very efficient, this strategy produced a code that could not be easily extended beyond *d*-type AOs. For this reason, here we have followed an alternative strategy for the generalization of the code to *f*- and *g*-type AOs (i.e., with l=3,4).

### 3.4. New Strategy Based on a Direct Evaluation of the Electron Density in the RSSH-GTF Basis

Instead of evaluating the electron density and its derivatives through an expansion of pair products of RSSH-GTFs in HGTFs as in Equation (16), we have devised a new strategy that works for l=0,1,2,3,4 (i.e., for *s*-, *p*-, *d*-, *f*-, and *g*-type AOs). The expressions for the AO pairs and their derivatives (up to order 4), required for evaluating the electron density and its derivatives in Equation (11), are directly evaluated in the RSSH-GTF basis, following Equation (2).

In a first step, we pre-calculated the symbolic expressions for the RSSH-GTF pairs from the computer algebra system for symbolic computation available in Matlab by exploiting the following two recurrence relations [52]:

(18) $$X_{n,l+1,\pm \left(l+1\right)}\left(r\right)=\left(2l+1\right)\left[r_{x}X_{n,l,\pm l}\left(r\right)\mp r_{y}X_{n,l,\mp l}\left(r\right)\right]\;,$$

and:

(19) $$X_{n,l+1,m}\left(r\right)=\frac{1}{l-|m|+1}\left[\left(2l+1\right)r_{z}X_{n,l,m}\left(r\right)-\left(l+|m|\right){∥r∥}^{2}X_{n,l-1,m}\left(r\right)\right]\;.$$

Equations (18) and (19) allow us to generate the RSSHs for the entire set of *l* and *m* values for a given *n* (here, we limit our considerations to n=0). The starting point for the recurrence is with the *s*-type RSSH for which $X_{0,0,0}=1$ and, in connection with Equation (18), the convention $X_{0,0,-0}=0$ is understood. The algorithm then proceeds with the calculation of $X_{0,l+1,l+1}$ and $X_{0,l+1,-(l+1)}$ through Equation (18) for l=1,2,3,4. The RSSH $X_{0,l,m}$ with $m\in \left[-l+1,l-1\right]$ are then generated for successively higher *l*. The steps of the used algorithm are summarized in the following, where quantum numbers in bold are being increased:

$$\left(1\right)X_{0,0,0}=1\;\left(2\right)X_{0,l,l}\;\left(3\right)X_{0,l,-l}\;\left(4\right)X_{0,1,m}\;\left(5\right)X_{0,l,m}$$

Once the full set of RSSH-GTF pairs were generated, the expressions for the derivatives of the RSSH-GTF pairs were determined by symbolic differentiation. Finally, we have generated Fortran routines containing the symbolic expressions for the (derivatives of the) RSSH-GTF pairs.

The new code has the obvious advantage of being generally applicable to basis sets with AOs of *s*-, *p*-, *d*-, *f*-, and *g*-type compared to the old one that was limited to work with *s*-, *p*- and *d*-type AOs. At the same time, it is found to be less efficient than the original one. For this reason, the old code is active by default for those systems where *f*-, and *g*-type AOs are not included in the basis set, with the new code coming into play only when needed (i.e., in the presence of *f*-, and *g*-type AOs in the basis set).

### 3.5. The Electron Localization Function

Besides ρ(r) and L(r), another scalar field which is commonly analyzed to extract useful information on the electron distribution is the electron localization function η(r) (ELF). Roughly speaking, the ELF function provides a (local) measure of electron localization in a system relative to that of an electron gas having the same electron density ρ(r) of the system [14]. The various, though largely compatible, definitions of η(r) share the general formula:

(20) $$\eta \left(r\right)=\frac{1}{\left[1+c{\left(r\right)}^{2}\right]}$$

where c(r) is the relevant kernel of the ELF and where η is scaled relative to *c* to bound its values between 0 and 1. ELF was first introduced by Becke and Edgecombe (BE) using as a kernel, $c_{B}E\left(r\right)$, the ratio of the curvatures of the spherically averaged same-spin conditional pair densities for the system under study and for the corresponding (i.e., same density) uniform electron gas [53]. Later on, Savin et al. proposed a more general and simpler definition, $c_{S}avin\left(r\right)=t_{p}\left(r\right)/t_{ph}\left(r\right)$, where $t_{p}\left(r\right)$ is the Pauli kinetic energy density of the system and $t_{ph}\left(r\right)$ that of the uniform electron gas with the same density [54]. The Pauli kinetic energy density represents the local increase of the kinetic energy due to the redistribution of the electrons caused by the Pauli principle; that is, the local kinetic energy excess relative to the kinetic energy density of a bosonic system. Use of either $c_{\mathrm{BE}}\left(r\right)$ or $c_{\mathrm{Savin}}\left(r\right)$ leads to formally equivalent ELF expressions, within the Hartree–Fock approximation originally adopted by BE. The interpretation due to Savin et al. has, however, a great conceptual advantage: regardless of the kind of adopted wave function, the same expression for the ELF is retained. In addition, being based on the kinetic energy density rather than on the electron pair density, the ELF can also easily be evaluated within the DFT, as in our case.

## 4. Results and Discussion

**Figure 1:**
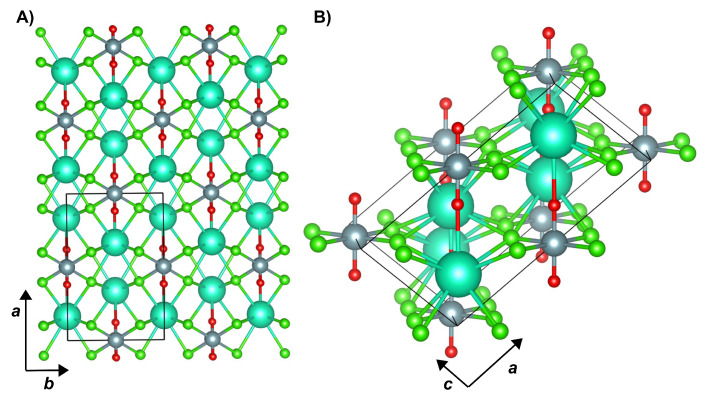
Structure of the cesium uranyl chloride, Cs${}_{2}$UO${}_{2}$Cl${}_{4}$, crystal viewed (**A**) in the ab crystallographic plane, and (**B**) down the *b* axis. Colors are as follows: O red, Cl, green, U grey, Cs, turquoise.

Cesium uranyl chloride, Cs${}_{2}$UO${}_{2}$Cl${}_{4}$, crystallizes in a monoclinic lattice with space group C2/m, whose atomic structure is shown in Figure 1. Each U atom forms two symmetry-equivalent bonds with O atoms (bond length of 1.776 Å in the experimental geometry) as well as four symmetry-equivalent bonds with Cl equatorial ligands (bond length of 2.670 Å). Each Cs atom is connected with eight Cl atoms (four symmetry-independent pairs with bond lengths in the range 3.502–3.624 Å) and one O atom (bond length of 3.259 Å). For a better comparison with the experimental electron density [18,19], we have performed our quantum-mechanical simulations on the experimental geometry. Calculations are performed both on the actual periodic model (here referred to as cry-Cs${}_{2}$UO${}_{2}$Cl${}_{4}$) and on an extracted molecular fragment [UO${}_{2}$Cl${}_{4}$]${}^{2-}$ to quantify the effect of the crystalline environment on the chemical bonding features of the U atom.

Table 1 reports computed atomic charges for the different species in the two systems (the extended crystal and the molecular fragment). Atomic charges are computed according to two schemes: a simple Mulliken orbital partitioning (M) and Bader’s integration of the electron density over atomic basins (B). The two approaches provide similar values for Cl and Cs while the QTAIMAC systematically results in larger charges for U and O, both in the molecular fragment and in the crystal. The computed atomic charges of the molecular fragment are in excellent agreement with those previously reported by Vallet et al. [38]. From inspection of the computed QTAIMAC charges on the extended crystal, we find that the predicted electronic structure of the system is very close to the limit ionic [Cs${}_{2}$]${}^{2+}$ [UO${}_{2}$Cl${}_{4}$]${}^{2-}$ one. When passing from the isolated molecular fragment to the actual periodic crystal, the most affected atomic charges are those of the U and O atoms, which both increase in absolute value. A closer inspection of the computed atomic charges already suggests a stronger polar character of the U–Cl bonds than the U–O ones. Indeed, each Cl is found to host an extra 0.74 electrons with respect to its neutral state for a total of 0.74 × 4 = 2.96 extra electrons hosted by the four Cl atoms surrounding the U atom. This value almost perfectly matches the atomic charge of U (+2.94), thus suggesting that the U atom donates about three electrons to the four Cl atoms while it participates in a much more covalent interaction with O. The agreement between our computed charges and those derived by the experiment is remarkable and corroborates this picture.

Chemical bonding features of cesium uranyl chloride have been analyzed by Pinkerton and co-workers by performing a QTAIMAC topological analysis of the experimentally-reconstructed electron density [18,19]. Table 2 reports several descriptors evaluated at the various bond critical points of the crystal from the experiments, along with the corresponding values we obtained from our quantum-mechanical calculations both on the actual periodic extended lattice (Cry) and on the extracted molecular fragment [UO${}_{2}$Cl${}_{4}$]${}^{2-}$ (Mol). Calculations are performed at the experimental geometry to ensure a more direct comparison. Overall, the two chemical bonds involving uranium (U–O and U–Cl) can both be classified [55] as of “incipient covalent” character (having 1<|V|/G<2, a negative total energy density H<0, and positive Laplacian $\nabla^{2}\rho >0$) but show very different chemical bonding features. In particular, the much higher covalent character of the U–O bond is clearly seen in terms of (i) the much larger absolute value of the bond degree |H|/ρ of 0.9 a.u. compared to 0.2 a.u. of U–Cl and (ii) the significantly larger value of the |V|/G ratio of about 1.8 compared to 1.3 of U–Cl. All chemical interactions involving Cs are very weak and of “closed-shell” type with |V|/G<1, a positive total energy density H>0, and a positive Laplacian $\nabla^{2}\rho >0$. The overall agreement between experimental and computed descriptors at the bond critical points is excellent for all the weak interactions (including some subtle aspects such as the Cs-O interaction being characterized by the lowest electron density at the bond CP among all interactions involving Cs), good for the U–Cl interaction and definitely less satisfactory for the U–O interaction. Indeed, as per the U–O bond, while some descriptors (such as the bond degree H/ρ) show a rather good agreement, others (such as the electron density and its Laplacian at the bond CP) show large discrepancies. This was previously noted [38], tentatively attributed to missing environmental effects, and finally shown not to depend on crystal field effects [39]. This latter conclusion is further corroborated by our present results in Table 2 where differences between the molecular fragment [UO${}_{2}$Cl${}_{4}$]${}^{2-}$ and the actual extended solid are seen to be very small if not negligible on both U–O and U–Cl interactions.

**Figure 2:**
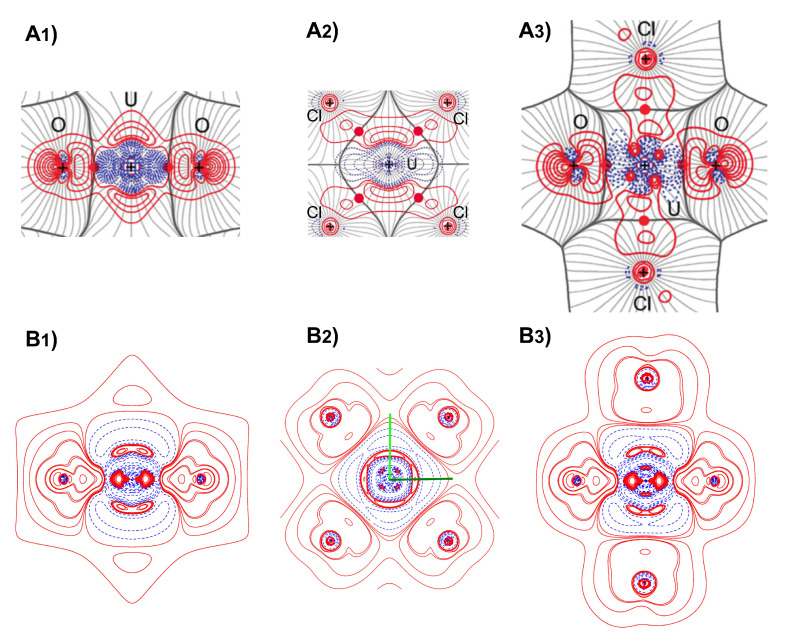
Deformation density, Δρ(r), contour maps of the cesium uranyl chloride crystal around the U atom in three different planes: (**left**) through the O–U–O axis and the **b** crystal lattice vector, (**center**) the equatorial plane of the four Cl atoms, (**right**) through the O–U–O axis and a pair of Cl atoms. Upper panels report the experimental deformation density from Ref. [19] while bottom panels report the computed one in this study. Contour values are ± 0.05, 0.15, 0.25, 0.4, 0.7, 1.0, 1.5, 2.0 e/Å${}^{-3}$. Red and blue lines correspond to positive and negative values, respectively. In the experimental upper panels, DD contour lines are overlaid over the ∇ρ trajectories of the crystal electron density.

In order to analyze further the origin of the discrepancy in the description of the U–O interaction between theory and experiments, we present deformation density (DD) maps in Figure 2. Deformation density (relative to a neutral atomic reference), Δρ(r), contour maps of the cesium uranyl chloride crystal around the U atom are shown in three different planes: (left) through the O–U–O axis and the **b** crystal lattice vector, (center) the equatorial plane of the four Cl atoms, (right) through the O–U–O axis and a pair of Cl atoms. Upper panels report the experimental deformation density from Ref. [19] while bottom panels report the computed one in this study. The same iso-values have been used for the computed and experimental contour maps. In the panels (A${}_{1}$–A${}_{3}$), DD contour lines are overlaid over the ∇ρ trajectories of the crystal electron density, so as to show the intersection of the atomic boundaries with the three considered planes. Both quantitative and qualitative differences can be seen in the description of the electron distribution in the bonding region around the U atom. Panels (A${}_{1}$) and (B${}_{1}$) both show the nearly axial symmetry of the U–O interactions. In particular, the DD of present quantum-mechanical calculations in panel (B${}_{1}$) corroborates [11,56,57] the previously suggested “triple bond” nature of the U–O interaction with a sp hybridization of the oxygen and the formation of a σ bond along the U–O axis and, supposedly, two π bonds with a maximum of charge deformation at about 0.71 Å off the axis. The charge accumulation close to the U atom along the U–O axis in the σ bond in panel (B${}_{1}$)—suggestive of a covalent character—is instead missing to a large extent in the experimental DD in panel (A${}_{1}$). Other qualitative differences are observed in (i) a higher degree of localized electron build-up in the region of π bonds in the theoretical DD and (ii) a more pronounced charge deformation behind the oxygen atoms in the experimental DD. Large differences are also observed in the equatorial plane of the four Cl atoms in panels (A${}_{2}$) and (B${}_{2}$). In particular, the expected nearly 4-fold symmetry of this plane seems to be lost in the experimental DD of panel (A${}_{2}$) while it is still largely there in the computed DD of panel (B${}_{2}$). The large departure from the expected symmetry was acknowledged in Ref. [19] and tentatively attributed to the different crystal environment at the second and third nearest neighbor level. While present calculations do show some asymmetry in the equatorial plane (for instance, inspection of the DD along the two green lines in panel (B${}_{2}$) reveals that those two directions are not exactly symmetry equivalent), they predict it to be very subtle while preserving the overall symmetric distribution of the density around the U atom in this plane. However, both theory and experiments describe a charge depletion close to U and a charge accumulation close to Cl along the U–Cl bonds, indicative of a higher ionic character of this interaction relative to the U–O one. Panels (A${}_{3}$) and (B${}_{3}$) show the DD in a plane through the O–U–O axis and a pair of Cl atoms and basically confirm all of the discrepancies of those features discussed above on the other two planes.

**Figure 3:**
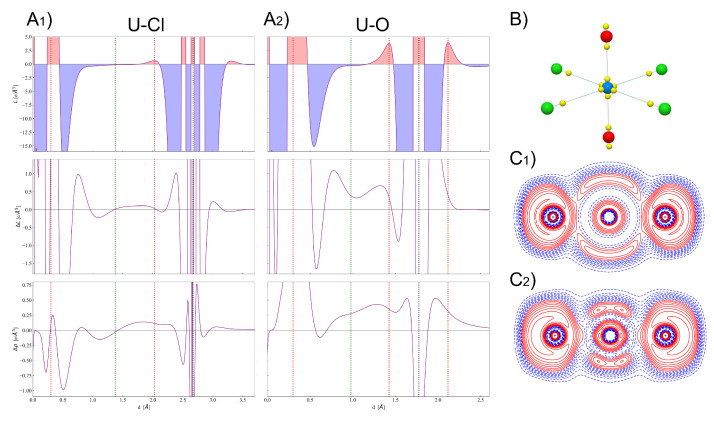
(**A**${}_{1}$) Laplacian function L(r) (upper panel), deformation Laplacian ΔL(r) (middle panel) and deformation density Δρ(r) (bottom panel) along the U–Cl axis. (**A**${}_{2}$) Same as in (**A**${}_{1}$) but along the U–O axis. Vertical dashed black lines mark the distance of Cl and O from U, vertical dashed green lines mark the position of the bond critical point and vertical dashed red lines mark the position of (3,+3) CPs of the Laplacian. (**B**) Spatial distribution of (3,+3) CPs of the Laplacian in the [UO${}_{2}$Cl${}_{4}$]${}^{2-}$ molecular fragment (yellow spheres). (**C**${}_{1}$) Electron localization function (ELF) in the region of the O–U–O bonds from a superposition of non-interacting atomic densities. (**C**${}_{2}$) Same as in (**C**${}_{1}$) but from the computed density of the crystal.

The analysis of the distribution of the electron density ρ(r) is often complemented by the analysis of its Laplacian $\nabla^{2}\rho \left(r\right)$ or, equivalently, of $L\left(r\right)=-\nabla^{2}\rho \left(r\right)$, which provides additional information on the spatial distribution of the electrons and in particular on the asphericity of (bonded) atoms [15]. In particular, some critical points of the Laplacian correspond to charge concentrations and depletions in the core and valence shells. Valence shell charge concentrations (VSCCs) are particularly relevant to the rationalization of chemical bonding, and can be identified in terms of critical points of L(r) of (3,−3) type (i.e., maxima). Figure 3B shows the position in space of the VSCCs critical points (yellow spheres) as obtained from our quantum-mechanical calculations on the [UO${}_{2}$Cl${}_{4}$]${}^{2-}$ molecular fragment. The number and position in space of the VSCCs around the U atom are very different with respect to those of the [UF${}_{6}$]${}^{-}$ molecule that we studied previously [37]. In that case, 14 VSCCs were found around U while here only 6, all of which lying along the axes of the bonds with Cl and O, thus indicating that charge concentration around U only occurs in the direction of the bonds. The radial distance from U of these 6 critical points is of 0.30 Å and corresponds to the maxima of the VSCC of the principal quantum number n= 6. Along the direction of the U–Cl bond, a critical point is found at 0.64 Å from Cl, which corresponds to the chlorine atom VSCC of n= 3. No other (3,-3) L(r) critical points are found behind the Cl atoms in the molecular fragment, thus indicating that their otherwise spherical density distribution is only altered along the Cl–U internuclear axis. Such an almost spherical electron distribution is broken in the crystal because of the weak interactions with the four Cs atoms surrounding each Cl, with the emergence of further critical points (see below). Finally, along the U–O axis, two critical points corresponding to the O valence charge concentrations are observed: one at 0.35 Å from O toward U and one at 0.34 Å behind O, which suggests a sp-like hybridization of O along the U–O direction. Such a feature could not be clearly seen from our computed DD maps in Figure 2 while it can be further corroborated by inspection of Figure 3C${}_{1}$,C${}_{2}$, where maps of the electron localization function (ELF) [58,59] in the region of the O–U–O bonds are shown. Panel (C${}_{1}$) reports the computed ELF from a superposition of non-interacting atomic densities while panel (C${}_{2}$) the ELF from the actual computed density of the interacting system. A η(r) value equal to 0.5 indicates electron localization similar to that of the uniform electron gas, while η(r) values greater or smaller than 0.5 (red and blue iso-contours in the figure) denote higher or lower electron localization compared to the uniform electron gas. The comparison of the two maps clearly shows the higher localization of the electrons both in the σ and π region of the U≡O triple bond and behind the O atoms.

**Figure 4:**
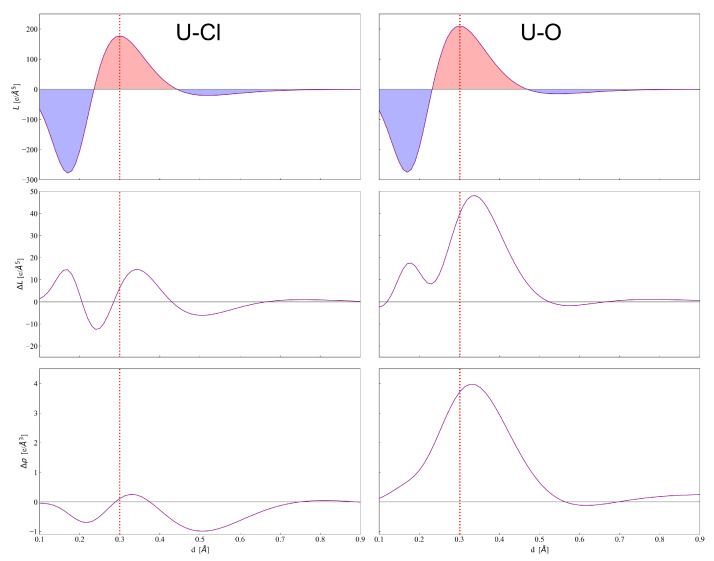
Laplacian function L(r) (upper panel), deformation Laplacian ΔL(r) (middle panel) and deformation density Δρ(r) (bottom panel) along the U–Cl and U–O axes in the vicinity of the U atom. Vertical dashed red lines mark the position of U VSCC CPs of the Laplacian.

Panels (A${}_{1}$) and (A${}_{2}$) of Figure 3 show the evolution of L(r) (upper panel), ΔL(r) (middle panel) and Δρ(r) (bottom panel) along the U–Cl and U–O axes, respectively, for a more quantitative analysis of the features of chemical bonding discussed above. Analogous panels, with a highly magnified interval range on the y-axis and reduced interval range on the x-axis, are reported in Figure 4 to show details of the same functions in the U atom VSCC region. These panels clearly show the difference between the highly polar U–Cl interaction and the definitely more electron shared U–O bond. The L(r) profile in the VSCC and VSCD (valence shell charge depletion) regions of U indicate that the former region is much larger and the latter much smaller when U is bonded to O rather than to Cl, and so are the magnitudes of the corresponding maxima and minima along the profile. In fact, the value of L(r) at the (3,-3) CP in the U VSCC is 177.2 and 210.8 e/Å${}^{5}$ for U–Cl and U–O bonding interactions, respectively, and the values of ρ(r) at this CP follow a similar increasing trend being 6.0 and 6.5 a.u., respectively. The different nature of the two interactions is confirmed by their ΔL(r) and Δρ(r) profiles in the region around the bond critical point (green vertical line in the profiles in Figure 3, with U–O showing a definitely larger charge concentration (larger ΔL(r)) and charge build-up (larger Δρ(r)) relative to the U–Cl case. Moreover, such distinct behaviors in the bonding region are observed in a much wider r interval for the U–O rather than for the U–Cl interaction. Finally, it is interesting to note that the two (3,-3) CPs in the VSCC of O are of similar nature (compatible with sp-like hybridization) but not perfectly equivalent. The one facing the U atom and directly involved in the U–O interaction is slightly more displaced from the O nucleus (0.35 Å versus 0.34 Å) and has a larger ρ(r) value (0.877 versus 0.862 a.u.) as a clear sign of (partially) covalent bonding. Moreover, a smaller L(r) value is observed at this (3,-3) CP (3.8 versus 3.9 e/Å${}^{5}$) since the associated VSCC region has expanded toward the facing U atom and become larger in size for promoting the (partially) covalent bonding interaction.

**Figure 5:**
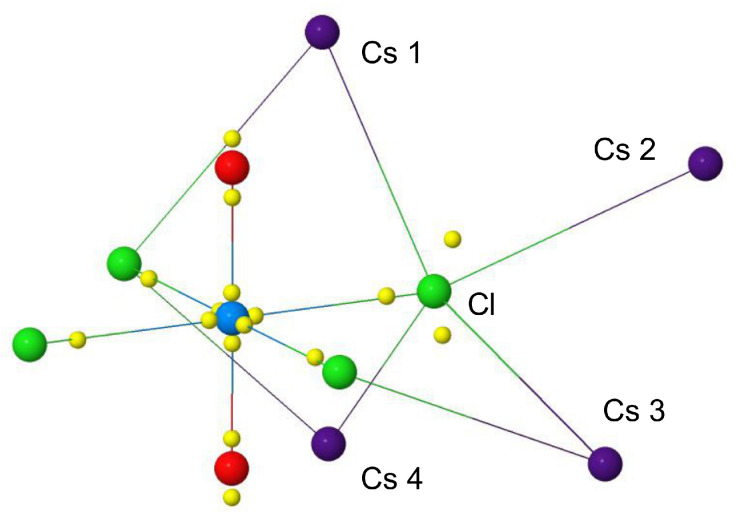
Positions in space of (3,+3) CPs of the Laplacian of the electron density of the cesium uranyl chloride (yellow spheres), as obtained from our quantum-mechanical periodic calculations. These CPs correspond to maxima of valence charge concentrations of U, O and Cl atoms.

As anticipated above, the electron distribution around the Cl atoms in the crystal gets perturbed by the weak interactions with the four inequivalent Cs atoms surrounding it. This breaking of an otherwise almost spherical distribution is well captured by the appearance of two extra CPs of (3,+3) type of the Laplacian of the electron density, as shown in Figure 5. These two CPs, corresponding to regions of valence charge concentration, are found along directions in between neighboring Cs atoms and not along Cl–Cs axes, denoting the presence of an ionic rather than a Cl–Cs dative interaction.

## 5. Conclusions and Perspectives

The Topond module (previously limited to work in terms of *s*-, *p*- and *d*-type basis functions only) of the Crystal program has been generalized to *f*- and *g*-type basis functions, within the linear combination of atomic orbitals (LCAO) approach. The main formal and computational aspects behind this generalization have been presented. The new algorithms now allow for an effective characterization of chemical bonding of materials containing lanthanides and actinides through a topological analysis of the electron density and its Laplacian.

The program has been applied to the analysis of chemical bonding of the cesium uranyl chloride, Cs${}_{2}$UO${}_{2}$Cl${}_{4}$, crystal. Discrepancies between the present quantum-mechanical description of the electron density distribution and that previously reconstructed by experimental X-ray diffraction are illustrated and the implications on the description of chemical bonding discussed.

As a next step, a collaborative study is planned with the researchers who ran the X-ray diffraction experiments and re-constructed the corresponding experimental electron density about 10 years ago to further analyze the observed discrepancies. Indeed, their multipolar model refinement strategy has significantly improved in recent years, which may lead to a closer agreement with present quantum-mechanical predictions.

## Figures and Tables

**Table 1:** Atomic charges computed on the actual periodic system cry-Cs${}_{2}$UO${}_{2}$Cl${}_{4}$ and on the extracted molecular fragment [UO${}_{2}$Cl${}_{4}$]${}^{2-}$. Both Mulliken (M) and QTAIMAC Bader (B) charges are reported. Experimental values derived from a QTAIMAC analysis of the reconstructed electron density of the crystal are also reported for comparison [19].

	[UO${}_{2}$Cl${}_{4}$]${}^{2-}$		cry-Cs${}_{2}$UO${}_{2}$Cl${}_{4}$		
	Q${}_{M}$	Q${}_{B}$	Q${}_{M}$	Q${}_{B}$	Q${}_{B,\exp}$
U	+2.18	+2.90	+2.14	+2.94	+2.75
O	−0.61	−0.93	−0.60	−1.02	−0.92
Cl	−0.74	−0.76	−0.74	−0.74	−0.60
Cs	-	-	+1.00	+0.98	+0.77

**Table 2:** Descriptors of chemical bonding of cesium uranyl chloride from the QTAIMAC: bond length *l*, distance between first atom and bond critical point $d_{\mathrm{CP}}$, value of several local quantities at the bond critical point such as the electron density ρ, the Laplacian of the density $\nabla^{2}\rho$, the ratio between the potential energy density and kinetic energy density |V|/G, and the bond degree H/ρ (i.e., ratio between total energy density and electron density). Calculations are performed at the experimental geometry. Experimental data are taken from Ref. [19]. Calculations are performed both on the actual periodic extended lattice (Cry) and on the extracted molecular fragment [UO${}_{2}$Cl${}_{4}$]${}^{2-}$ (Mol).

		O–U	Cl–U	O–Cs	Cl–Cs	Cl–Cs	Cl–Cs	Cl–Cs
*l* (Å)	Exp	1.776	2.670	3.259	3.502	3.518	3.544	3.624
	Mol	0.797	1.298	-	-	-	-	-
$d_{\mathrm{CP}}$ (Å)	Cry	0.797	1.298	1.458	1.693	1.715	1.734	1.773
	Exp	0.818	1.279	1.494	1.729	1.703	1.757	1.771
	Mol	2.059	0.421	-	-	-	-	-
ρ (*e*/Å${}^{3}$)	Cry	2.058	0.425	0.057	0.070	0.069	0.066	0.058
	Exp	1.695	0.486	0.053	0.067	0.071	0.076	0.068
	Mol	7.47	3.44	-	-	-	-	-
$\nabla^{2}\rho$ (*e*/Å${}^{5}$)	Cry	7.55	3.42	0.88	0.81	0.79	0.76	0.65
	Exp	15.77	3.28	0.72	0.65	0.73	0.64	0.63
	Mol	1.780	1.256	-	-	-	-	-
|V|/G	Cry	1.778	1.264	0.740	0.799	0.825	0.823	0.806
	Exp	1.587	1.417	0.724	0.845	0.831	0.902	0.860
	Mol	−0.903	−0.196	-	-	-	-	-
H/ρ (a.u.)	Cry	−0.902	−0.202	0.224	0.141	0.119	0.121	0.128
	Exp	−0.926	−0.340	0.204	0.091	0.105	0.053	0.079

## Data Availability

Data are contained within the article and supplementary material.

## Supplementary Materials

The following are available online, The input file for the Crystal calculation of the Cs${}_{2}$UO${}_{2}$Cl${}_{4}$ crystal.
