# Supplementary material for: Topology of the Electron Density and of Its Laplacian from Periodic LCAO Calculations on f-Electron Materials: The Case of Cesium Uranyl Chloride
Source: Molecules. 2021 Jul 12;26(14):4227. doi: 10.3390/molecules26144227 (PMC8303866; doi:10.3390/molecules26144227)
Supplement: Supplementary file 1 [file molecules-26-04227-s001.zip › molecules-1259661-supplementary.pdf]

Supporting Information: Topology of the Electron Density and of its Laplacian from Periodic LCAO Calculations on f-Electron Materials: The Case of Cesium Uranyl Chloride

Alessandro Cossard,† Silvia Casassa,† Carlo Gatti,‡ Jacques K. Desmarais,*,† and Alessandro Erba*,†

†*Dipartimento di Chimica, Università di Torino, via Giuria 5, 10125 Torino, Italy*

‡*CNR-SCITEC, Istituto di Scienze e Tecnologie Chimiche "Giulio Natta", via C. Golgi 19, 20133 Milano, Italy*

E-mail: jacqueskontak.desmarais@unito.it; alessandro.erba@unito.it

1 Crystal Input for $Cs_2UO_2Cl_4$

```
CS2UO2Cl4

CRYSTAL

0 0 0

12

11.78820 7.64110 5.76860 100.4380

4

292   0.00000    0.00000    0.00000

255   0.66487    0.00000    0.32067

17   0.39809    0.24695    0.20704

8    0.11906    0.00000    0.23790

END

292 31

INPUT

32. 0 4 8 8 8 6

16.91870874 529.53526911 0

3.40970576 4.27018845 0

0.79302733 0.09998874 0

0.19378381 0.00626781 0

13.16953414 100.93359134 0

10.60784728 175.95423897 0

2.69049397 -0.00210787 0

2.08929800 -0.19041648 0

0.54050990 0.00494627 0

0.40482776 -0.01652483 0

0.11250285 0.00082033 0

0.09508873 -0.00100028 0

9.06784123 62.85927902 0
```

```
8.53362678 90.20882494 0
1.63646790 -0.08282418 0
1.54425719 -0.15307917 0
0.47961552 -0.00008720 0
0.41164502 0.00484078 0
0.13990510 -0.00006136 0
0.17494682 -0.00240839 0
5.14746012 15.68628229 0
5.29241394 22.32105345 0
1.05726701 -0.20689333 0
0.98063114 -0.08434451 0
0.48259555 0.06084446 0
0.55434882 0.00231264 0
0.23674544 -0.00204069 0
0.21559852 0.00348388 0
18.83643086 -44.41029420 0
18.74850924 -53.65339478 0
6.49279545 -2.55219343 0
6.57472519 -3.34380088 0
2.58151924 0.04527524 0
2.58690949 0.05637947 0
0 0 1 2 1
  29520.834 1.
0 0 1 2 1
  4449.8874 1.
0 0 1 2. 1
  1018.7754 1.
0 0 1 0 1
  289.5348 1.
```

```
0 0 1 0 1
  46.9990 1.
0 0 1 0 1
  23.9791 1.
0 0 1 0 1
  10.1441 1.
0 0 1 0 1
  2.7658 1.
0 0 1 0 1
  0.6151 1.
0 0 1 0 1
  0.2780 1.
0 2 1 6 1
  499.7488 1.
0 2 1 6 1
  114.0192 1.
0 2 1 0 1
  15.6492 1.
0 2 1 0 1
  7.9843 1.
0 2 1 0 1
  3.1325 1.
0 2 1 0 1
  1.6077 1.
0 2 1 0 1
  0.7061 1.
0 2 1 0 1
  0.3229 1.
0 3 1 10 1
```

```
  75.1703 1.
0 3 1 1 1
  20.7869 1.
0 3 1 0 1
  5.5167 1.
0 3 1 0 1
  2.6058 1.
0 3 1 0 1
  1.2781 1.
0 3 1 0 1
  0.5617 1.
0 3 1 0 1
  0.2135 1.
0 4 1 3. 1
  8.1761 1.
0 4 1 0 1
  3.5111 1.
0 4 1 0 1
  1.6789 1.
0 4 1 0 1
  0.7604 1.
0 4 1 0 1
  0.3170 1.
0 5 1 0 1
  0.7604 1
255 3
HAYWSC
0 1 3 8 1
2.7869668567 0.0406474598702 -0.0165526980724
```

```
1.15910578043 -0.555331349468 -0.156354595401
0.444885148239 0.790078910046 0.653388515688
0 1 1 1 1
0.152606321165 1. 1.
0 3 1 0 1
0.599990264849 1.
17 13
0 0 7 2 1
  69507.9909450             0.54314897497D-03
  10426.1568800             0.41990463961D-02
   2373.2334061             0.21592141679D-01
    671.56420071            0.84598850094D-01
    218.41999790            0.24757249724
     77.572249714           0.47016930228
     28.888815277           0.37436370716
0 0 3 2 1
    127.10527185            0.25182166603D-01
     39.339582961           0.10786112456
      7.6740679989         -0.27408821574
0 0 2 2 1
      3.8745627630          1.3213875014
      1.8385832573          0.68636955368
0 0 1 0 1
      0.50229057542         1.0000000
0 0 1 0 1
      0.17962723420         1.0000000
0 2 5 6 1
    666.50423284            0.23632663836D-02
    157.64241690            0.18879300374D-01
```

```
     50.262520978            0.87206341273D-01
     18.536078105            0.25285612970
      7.2940532777           0.43507154820
0 2 1 5 1
      2.9433248995            .35026513165
0 2 1 0 1
      1.0404970818           1.0000000
0 2 1 0 1
      0.38456415080          1.0000000
0 2 1 0 1
      0.13069642732          1.0000000
0 3 2 0 1
      4.61000000             0.20000000
      1.01100000             1.00000000
0 3 1 0 1
      0.339000000            1.0000000
0 4 1 0 1
      0.706000000            1.0000000
8 11
0 0 6 2 1
  27032.3826310              0.21726302465D-03
   4052.3871392              0.16838662199D-02
    922.32722710             0.87395616265D-02
    261.24070989             0.35239968808D-01
     85.354641351            0.11153519115
     31.035035245            0.25588953961
0 0 2 2 1
     12.260860728            0.39768730901
      4.9987076005           0.24627849430
```

```
0 0 1 0 1
      1.1703108158            1.0000000
0 0 1 0 1
      0.46474740994           1.0000000
0 0 1 0 1
      0.18504536357           1.0000000
0 2 4 4 1
     63.274954801             0.60685103418D-02
     14.627049379             0.41912575824D-01
      4.4501223456            0.16153841088
      1.5275799647            0.35706951311
0 2 1 0 1
      0.52935117943            .44794207502
0 2 1 0 1
      0.17478421270            .24446069663
0 3 1 0 1
      2.31400000              1.0000000
0 3 1 0 1
      0.64500000              1.0000000
0 4 1 0 1
      1.42800000              1.0000000
99 0
END
DFT
XXLGRID
B3LYP
ENDDFT
TOLINTEG
8 8 8 8 20
```

```
MAXCYCLE
200
FMIXING
95
SMEAR
0.0001
SHRINK
6 6
TOLDEE
9
PPAN
END
```
